# A Patient-Reported Outcome Tool to Triage Total Hip Arthroplasty Patients to Hospital or Video Consultation: Pilot Study With Expert Panels and a Cohort of 1228 Patients

**DOI:** 10.2196/31232

**Published:** 2021-12-20

**Authors:** Yvette Pronk, Peter Pilot, Walter van der Weegen, Justus-Martijn Brinkman, Berend Willem Schreurs

**Affiliations:** 1 Research Department Kliniek ViaSana Mill Netherlands; 2 IMUKA Roosteren Netherlands; 3 Department of Orthopaedic Surgery Anna Ziekenhuis Geldrop Netherlands; 4 Department of Orthopaedic Surgery Kliniek ViaSana Mill Netherlands; 5 Department of Orthopaedic Surgery Radboud University Medical Center Nijmegen Netherlands

**Keywords:** PROMs, total hip arthroplasty, triage tool, video consultation, telemedicine, digital transformation

## Abstract

**Background:**

The digital transformation in health care has been accelerated by the COVID-19 pandemic. Video consultation has become the alternative for hospital consultation. It remains unknown how to select patients suitable for video consultation.

**Objective:**

This study aimed to develop a tool based on patient-reported outcomes (PROs) to triage total hip arthroplasty (THA) patients to hospital or video consultation.

**Methods:**

A pilot study with expert panels and a retrospective cohort with prospectively collected data from 1228 THA patients was executed. The primary outcome was a PRO triage tool to allocate THA patients to hospital or video consultation 6 weeks postoperatively. Expert panels defined the criteria and selected the patient-reported outcome measure (PROM) questions including thresholds. Data were divided into training and test cohorts. Distribution, floor effect, correlation, responsiveness, PRO patient journey, and homogeneity of the selected questions were investigated in the training cohort. The test cohort was used to provide an unbiased evaluation of the final triage tool.

**Results:**

The expert panels selected moderate or severe pain and using 2 crutches as the triage tool criteria. PROM questions included in the final triage tool were numeric rating scale (NRS) pain during activity, 3-level version of the EuroQol 5 dimensions (EQ-5D-3L) questions 1 and 4, and Oxford Hip Score (OHS) questions 6, 8, and 12. Of the training cohort, 201 (201/703, 28.6%) patients needed a hospital consultation, which was statistically equal to the 150 (150/463, 32.4%) patients in the test cohort who needed a hospital consultation (*P*=.19).

**Conclusions:**

A PRO triage tool based on moderate or severe pain and using 2 crutches was developed. Around 70% of THA patients could safely have a video consultation, and 30% needed a hospital consultation 6 weeks postoperatively. This tool is promising for selecting patients for video consultation while using an existing PROM infrastructure.

## Introduction

The digital transformation in health care has been accelerated by the COVID-19 pandemic. Health care institutions are challenged by precautionary measures to contain COVID-19 while continuing to provide health care. Especially, managing the physical flow of patients is challenging. After the pandemic, hospitals will begin to eliminate their waiting lists while maintaining a normal patient flow, and they will be challenged again. As a solution, video consultation has become an alternative to the traditional hospital consultation. The number of hospital consultations has dropped by 30%, and the number of telemedicine visits has increased 5-fold [1].

Currently, video consultation provides health care institutions and clinicians the opportunity to increase office efficacy and cost-effectiveness in an era of decreasing reimbursements and increasing time constraints [2-4]. From the patient’s perspective, it can also improve care efficacy and patient satisfaction as well as eliminating travel time and expenses [1,2]. However, not all patients might benefit from video consultation, and it is unknown how to select patients suitable for video consultation.

Orthopedic associations in many countries advise hospitals to collect patient-reported outcomes (PROs) of total hip arthroplasty (THA) using selected patient-reported outcome measures (PROMs) to evaluate health care and improve patient care [5,6]. To prevent extra burden in time and costs, it would be efficient to apply these PROs to select which patients need a hospital consultation and who can have a video consultation instead. Therefore, the aim of this study was to develop a tool based on PROs to triage THA patients to hospital or video consultation 6 weeks postoperatively. It was hypothesized that 10% of the THA patients would need a hospital consultation, as around 90% of the performed THAs result in a favorable outcome [7-9].

## Methods

### Overview

A pilot study with expert panels and a retrospective cohort with prospectively collected data from THA patients was performed. Regarding the cohort, patients were included in this study if they signed the informed consent form preoperatively to allow further scientific analysis using their anonymized data. Therefore, the institutional review board ruled that formal approval was not required for this study. There were no exclusion criteria.

### Outcomes

The primary outcome was a PRO triage tool to allocate THA patients to a hospital consultation or a video consultation for their 6-week postoperative consultation. Hospital consultation was defined as needing a physical examination or other examination, such as an X-ray, for which a patient really needed to be in the hospital. If no hospital consultation was needed, patients were allocated to a video consultation. According to the Dutch guidelines, patients should be seen 6 to 12 weeks after a THA [10], which is mostly held at 6 weeks. As it is advised to collect the first postoperative PROs at 3 months and not at 6 weeks [6], the 3-month PROs were considered the most appropriate for this study. Based on previous studies, the assumption was made that there are limited clinically relevant differences between PROs at 6 weeks and 3 months postoperatively [11,12].

### Measurements

Measurements were divided into 3 parts: (1) expert panels defined the criteria and selected the PROM questions, including the thresholds; (2) investigation of the clinimetric qualities of selected questions or triage criteria groups in the retrospective cohort with prospectively collected data; and (3) evaluation of the final triage tool.

#### Selection by Expert Panels

Two expert panels were created: clinical expert panel and research expert panel. The clinical expert panel consisted of 4 high-volume THA orthopedic surgeons from 2 different health care institutions. The research expert panel consisted of 3 researchers from 2 different health care institutions. As step one, the clinical expert panel defined the clinical triage criteria for the triage tool. These clinical triage criteria were based on the clinical disabilities for which patients needed to have a physical examination or other examination, such as an X-ray. As the second step, based on the clinical triage criteria, the research expert panel selected the appropriate PROM questions, including the thresholds, based on previous studies. As step three, these questions and their thresholds were presented to the clinical expert panel to discuss if these questions and/or thresholds covered the clinical triage criteria. If no threshold was reported in previous studies, the threshold was set using clinical reasoning by the clinical expert panel. These steps resulted in the PROM questions, including the thresholds, that were determined to be clinically relevant for the triage tool.

#### Clinimetric Qualities of Selected Questions or Triage Criteria Groups

The retrospective cohort with prospectively collected data consisted of patients who underwent surgery between January 2016 and December 2018 in a medium-sized orthopedic hospital (Kliniek ViaSana, Mill, The Netherlands). Therefore, patients were characterized by an American Society of Anesthesiologists (ASA) score of I-II and BMI ≤35. Four high-volume, experienced orthopedic surgeons performed the primary posterolateral THAs. Length of stay was generally 1 or 2 days.

The data included patient characteristics, PROM response rates, and PROs. Patient characteristics were age on the day of surgery, gender, preoperative BMI, ASA scores, and preoperative Charnley scores collected from the electronic patient records. Response rates were calculated as the number of returned questionnaires that were partially or totally completed divided by the number of THAs minus the number of THAs of patients who were deceased (returned questionnaires / [THAs - THAs of patients who were deceased]) [5]. PROs were primary digitally collected (OnlinePROMs, Rosmalen, The Netherlands). If patients were unable to handle a computer, paper questionnaires were sent. A maximum of 2 reminders to complete the PROMs were sent [13]. PROs were collected preoperatively and 3 and 12 months postoperatively according to the advice of the Dutch Orthopedic Association. This advice included the following questionnaires: numeric rating scale (NRS) pain at rest, NRS pain during activity, 3-level version of the EuroQol 5 dimensions (EQ-5D-3L), Hip disability and Osteoarthritis Outcome Score – Physical Function Shortform (HOOS-PS), Oxford Hip Score (OHS), and an anchor question about functional improvement [6].

Pain at rest and pain during activity were both measured using an NRS question scored from 0 (no pain) to 10 (severe pain). Quality of life was assessed using the EQ-5D-3L questionnaire consisting of 2 parts: EQ visual analogue scale (EQ VAS; 0-100, with 0 as the worst imaginable health state and 100 as the best imaginable health state) and EQ-5D descriptive system existing of 5 questions about 5 dimensions. These 5 dimensions are mobility, self-care, usual activities, pain/discomfort, and anxiety/depression, scored from 1 (no problems) to 3 (extreme problems) [14]. Furthermore, hip function was measured using the HOOS-PS questionnaire, on a scale from 0 (no difficulty) to 100 (extreme difficulty). This questionnaire consists of 5 questions scored from 0 (no difficulty) to 5 (extreme difficulty) [15,16]. Hip function and pain were assessed using the OHS questionnaire, with scores ranging from 0 (most severe symptoms) to 48 (least symptoms). This questionnaire consists of 12 questions scored from 0 (no difficulty) to 4 (extreme difficulty) [17]. Moreover, functional improvement was inquired on a 7-point Likert scale question ranging from 1 (very much deteriorated) to 7 (very much improved).

Regarding the investigation of the clinimetric qualities of selected questions or triage criteria groups by the expert panels, the cohort was divided into 2 groups: training cohort of patients who underwent surgery in 2016 or 2017 and a test cohort of patients who underwent surgery in 2018. To assess which questions were appropriate for the triage tool, the following clinimetric qualities were investigated per selected question in the training cohort: distribution, floor effect, correlation, responsiveness, and PRO patient journey. PRO patient journey was defined as a change in recovery over time. Regarding distribution, if the question did not show any distinction (median and IQR on the same level), the question was found not to be an appropriate question for the triage tool. For floor effect, if more than 15% of the patients scored the worst score [18], the question had a problem with the floor effect and was not an appropriate triage tool question. Investigating correlation, if the question was correlated (*r*≥.7) with another selected question(s) [19], this question or one of the other(s) could be chosen instead of all the questions for the triage tool. Regarding responsiveness, if a question was not responsive (*P*>.05) [20,21], it did not distinguish well between clinical relevance and lack of clinical relevance and was not included in the tool. Furthermore, the PRO patient journeys of patients with a worse score and of patients with a better score than the threshold were investigated. If patients with a worse score on a question at 3 months scored well on that question at 12 months, this question was not included in the triage tool. To assess which questions within the selected triage criteria group (for example pain) were appropriate for the triage tool, homogeneity was investigated per triage criteria group in the training cohort. If the homogeneity increased by removing a certain question from this group (Cronbach α>.7) [18,19], this question did not fit in this group and could be removed from the triage tool.

#### Evaluation of the Final Triage Tool

The final triage tool was applied in the test cohort to provide an unbiased evaluation of the final tool fitted on the training dataset. Results in both cohorts were compared to investigate the hypothesis.

### Statistical Analysis

Results are reported as mean (SD), median (IQR), or n (%) based on the test performed. To investigate if there was any difference in patient characteristics, response rates, and preoperative PROs between the training and test cohorts, continuous variables were first checked for a normal distribution. Second, independent *t* tests or Mann-Whitney *U* tests for continuous variables were executed depending on the distribution of the data, and Pearson chi-square or Fisher exact tests were executed for categorical variables.

Distribution was investigated with a boxplot distribution, floor effect was determined by calculating the percentage of patients with a minimum score, and correlation was assessed with Spearman correlation analyses. Responsiveness was evaluated by performing Wilcoxon signed rank tests on the change in preoperative and 3-month scores [20,21]. The PRO patient journey of patients with a worse or better score than the threshold on a question at 3 months was evaluated by boxplot distribution at 12 months. Homogeneity was investigated with a reliability analysis, including “scale if item deleted.” Before this analysis was executed, NRS pain and EQ-5D-3L questions were recoded to the same direction as the OHS questions.

Finally, the triage tool was applied for both training and test cohorts. To test the hypothesis, the numbers of hospital and video consultations for both cohorts were compared using Pearson chi-square or Fisher exact tests.

An α of .05 was considered statistically significant. Statistical analyses were performed using SPSS version 25.0 (IBM Corp, Armonk, NY).

## Results

### Selection by Expert Panels

“Having moderate or severe pain” and “using 2 crutches” were defined as the triage criteria by the clinical expert panel (step 1). For the criterion of “having moderate or severe pain,” the research expert panel selected the following PROM questions: NRS pain at rest, NRS pain during activities, EQ-5D-3L question 4, and OHS questions 1, 8, 10, and 12. For both NRS pain questions, previous studies reported thresholds of ≤3 for no or mild pain and >3 for moderate to severe pain [22,23]. For the criterion of “using 2 crutches,” EQ-5D-3L question 1 and OHS question 6 were selected (step 2). The clinical expert panel assessed the selected questions, even the NRS pain question thresholds, as appropriate. The other thresholds were discussed and defined (step 3; Table 1).

**Table 1:** Triage criteria, selected clinically relevant questions, and defined thresholds by the expert panels.

Triage criterion and selected PROM^a^ question			PROM question (score range)		Defined threshold
**Having moderate or severe pain**					
	NRS^b^ pain at rest	How much pain from your hip (surgery side) did you experience at rest in the last week? (0-10)		≥4	
	NRS pain during activity	How much pain from your hip (surgery side) did you experience during activity in the last week? (0-10)		≥4	
	EQ-5D-3L^c^ question 4	Pain/discomfort (1-3)		≥3 (extreme pain)	
	OHS^d^ question 1	During the past 4 weeks...How would you describe the pain you usually had from your hip? (0-4)		≤1 (moderate or severe)	
	OHS question 8	During the past 4 weeks...After a meal (sitting at a table), how painful has it been for you to stand up from a chair because of your hip? (0-4)		≤2 (moderate, very, unbearable)	
	OHS question 10	During the past 4 weeks...Have you had any sudden, severe pain — “shooting,” “stabbing,” or “spasms” — from the affected hip? (0-4)		≤1 (most or every)	
	OHS question 12	During the past 4 weeks...Have you been troubled by pain from your hip in bed at night? (0-4)		≤2 (3 or 4, 5 or 6, all)	
**Using 2 crutches**					
	EQ-5D-3L question 1	Mobility (1-3)		≥3 (confined to bed)	
	OHS question 6	During the past 4 weeks...For how long have you been able to walk before pain from your hip becomes severe (with or without a stick)? (0-4)		≤2 (5-15 minutes, around the house only, not at all)	

^a^PROM: patient-reported outcome measure.

^b^NRS: numeric rating scale.

^c^EQ-5D-3L: 3-level version of the EuroQol 5 dimensions.

^d^OHS: Oxford Hip Score.

### Clinimetric Qualities of Selected Questions or Triage Criteria Groups

Response rates were statistically significantly equal between both training (n=746) and test (n=482) cohorts preoperatively (745/746, 99.9% versus 482/482, 100%; *P*=.99) and at 3 months (703/746, 94.2% versus 463/482, 96.1%; *P*=.24) and 12 months (693/746, 92.9% versus 457/482, 94.8%; *P*=.29) postoperatively. The training cohort consisted of significantly fewer patients than in the test cohort with an ASA I score (399/746, 53.5% versus 287/482, 59.5%; *P*=.04), lower Charnely scores (*P*=.048), higher preoperative HOOS-PS scores (median 46.1, IQR 37.7-55.9 versus median 46.1, IQR 33.9-55.9; *P*=.01), and lower preoperative OHS scores (median 24.0, IQR 19.0-29.0 versus median 25.0, IQR 19.0-31.0; *P*=.03; Table 2). The clinical expert panel assessed these differences as not clinically relevant to correct for.

**Table 2:** Characteristics of training and test cohorts.

Characteristics				Training cohort (n=746)		Test cohort (n=482)		*P* value
**Response rate, n (%)**								
	Preoperative		745 (99.9)		482 (100)		.99	
	3 months postoperative		703 (94.2)		463 (96.1)		.24	
	12 months postoperative		693 (92.9)		457 (94.8)		.29	
**Patient characteristics**								
	Age (years), median (IQR)		66.5 (61.0-72.0)		66.0 (60.0-72.0)		.73	
	Gender (male), n (%)		295 (39.5)		206 (42.7)		.27	
	BMI (kg/m^2^), median (IQR)		26.1 (24.1-28.4)		26.3 (24.1-28.5)		.48	
	ASA^a^ score – I, n (%)		399 (53.5)		287 (59.5)		.04	
	**Charnley score:**						.048	
		One hip affected by OA^b^, n (%)	163 (21.8)		108 (22.4)			
		Both hips affected by OA, n (%)	309 (41.4)		196 (40.7)			
		Contralateral hip OA, n (%)	163 (21.8)		82 (17.0)			
		Multiple joints affected by OA, n (%)	111 (14.9)		96 (19.9)			
**Preoperative PROs^c^, median (IQR)**								
	NRS^d^ pain at rest score		6.0 (4.0-7.0)		6.0 (3.8-7.0)		.18	
	NRS pain during activity score		8.0 (7.0-9.0)		8.0 (7.0-8.0)		.13	
	HOOS-PS^e^ score		46.1 (37.7-55.9)		46.1 (33.9-55.9)		.01	
	EQ-5D descriptive system^f^		0.693 (0.298-0.775)		0.693 (0.569-0.775)		.16	
	EQ VAS^g^		76.0 (63.3-89.8)		77.0 (60.0-86.3)		.82	
	OHS^h^		24.0 (19.0-29.0)		25.0 (19.0-31.0)		.03	

^a^ASA: American Society of Anesthesiologists.

^b^OA: osteoarthritis.

^c^PROs: patient-reported outcomes.

^d^NRS: numeric rating scale.

^e^HOOS-PS: Hip disability and Osteoarthritis Outcome Score — Physical Function Shortform.

^f^EQ-5D descriptive system: EuroQol 5 dimensions descriptive system.

^g^EQ VAS: EuroQol visual analogue scale.

^h^OHS: Oxford Hip Score.

Regarding the questions or triage criteria groups selected by the expert panels (Table 1), OHS question 10 showed no distribution. For floor effect, <15% of patients scored the minimum score on all questions separately. All questions were significantly correlated with each other (*P*<.001). Regarding correlations ≥0.7, NRS pain during activity correlated with NRS pain at rest (*r*=0.659, *P*<.001) and OHS question 1 (*r*=–0.676, *P*<.001; Table 3). Furthermore, all questions were shown to be responsive (*P*<.001; Table 4). Regarding the PRO patient journey, patients with a worse score than the threshold also reported worse scores at 12 months than patients with a better score than the threshold. Only one patient with a better score than the threshold on EQ-5D-3L question 1 at 3 months had a 12-month score (Table 5). The other questions included ≥11 patients below or above the threshold. Regarding homogeneity, a Cronbach α of 0.818 was found for the triage criteria group “pain.” When one of the questions in this group was removed, the Cronbach α was maintained at above 0.7. The triage criteria group “crutches” scored a Cronbach α of 0.628. As there were 2 questions in this group, none of them could be removed to investigate the Cronbach α.

**Table 3:** Distribution, floor effect, and correlation per selected patient-reported outcome measure (PROM) question.

PROM^a^ question	Distribution, median (IQR)	Floor effect, n (%)	Correlations		
			Correlations (*r*)^b^	Correlated question	*P* value
NRS^c^ pain at rest	0 (0-1)	3^d^ (0.4)	0.659	NRS pain during activity	<.001
NRS pain during activity	2 (0-3)	4^d^ (0.6)	0.659; –0.676	NRS pain at rest; OHS^e^ question 1	Both <.001
EQ-5D-3L^f^ question 4	1 (1-2)	13^g^ (1.9)	None	N/A^h^	N/A
OHS question 1	3 (3-4)	6^g^ (0.9)	–0.675	NRS pain during activity	<.001
OHS question 8	3 (3-4)	0^g^ (0.0)	None	N/A	N/A
OHS question 10	4 (4-4)	2^i^ (0.3)	None	N/A	N/A
OHS question 12	4 (3-4)	32^i^ (4.6)	None	N/A	N/A
EQ-5D-3L question 1	1 (1-2)	2^i^ (0.3)	None	N/A	N/A
OHS question 6	4 (3-4)	3^j^ (0.4)	None	N/A	N/A

^a^PROM: patient-reported outcome measure.

^b^Statistically significant correlations >0.6 or <–0.6 are presented.

^c^NRS: numeric rating scale.

^d^n=703.

^e^OHS: Oxford Hip Score.

^f^EQ-5D-3L: 3-level version of the EuroQol 5 dimensions.

^g^n=693.

^h^N/A: not applicable.

^i^n=694.

^j^n=690.

**Table 4:** Responsiveness for each patient-reported outcome measure (PROM) question.

PROM question	Preoperative, median (IQR)	3 months postoperative, median (IQR)	*P* value
NRS^a^ pain at rest	6 (4-7)	0 (0-1)	<.001
NRS pain during activity	8 (7-9)	2 (0-3)	<.001
EQ-5D-3L^b^ question 4	2 (2-3)	1 (1-2)	<.001
OHS^c^ question 1	1 (0-1)	3 (3-4)	<.001
OHS question 8	2 (2-3)	3 (3-4)	<.001
OHS question 10	2 (1-3)	4 (4-4)	<.001
OHS question 12	2 (0-3)	4 (3-4)	<.001
EQ-5D-3L question 1	2 (2-2)	1 (1-2)	<.001
OHS question 6	2 (2-3)	4 (3-4)	<.001

^a^NRS: numeric rating scale.

^b^EQ-5D-3L: 3-level version of the EuroQol 5 dimensions.

^c^OHS: Oxford Hip Score.

**Table 5:** Patient journey per patient-reported outcome measure (PROM) question.

PROM question	Defined threshold	12-month score of patients with a score below the threshold at 3 months, median (IQR)	12-month score of patients with a score above the threshold at 3 months, median (IQR)
NRS^a^ pain at rest	≥4	0 (0-1)	2 (0-5)
NRS pain during activity	≥4	0 (0-1)	2 (0-4)
EQ-5D-3L^b^ question 4	≥3	1 (1-2)	2 (1.5-2)
OHS^c^ question 1	≤1	3 (2-4)	4 (3-4)
OHS question 8	≤2	3 (3-4)	4 (4-4)
OHS question 10	≤1	4 (2.5-3)	4 (4-4)
OHS question 12	≤2	3 (1.5-4)	4 (4-4)
EQ-5D-3L question 1	≥3	1 (1-2)	1 (1-1)^d^
OHS question 6	≤2	3 (2-4)	4 (4-4)

^a^NRS: numeric rating scale.

^b^EQ-5D-3L: 3-level version of the EuroQol 5 dimensions.

^c^OHS: Oxford Hip Score.

^d^n=1.

Based on the clinimetric qualities of selected questions or triage criteria groups, NRS pain at rest, OHS question 1, and OHS question 10 were removed from the triage tool. The final triage tool consisted of NRS pain during activity; EQ-5D-3L questions 1 and 4; and OHS questions 6, 8, and 12.

### Evaluation of the Final Triage Tool

The final triage tool resulted in 201 (201/703, 28.6%) patients in the training cohort needing a hospital consultation, which was statistically equal to the 150 (150/463, 32.4%) patients in the test cohort who needed a hospital consultation (*P*=.19).

## Discussion

This study aimed to develop a tool based on PROs collected using an existing PROM infrastructure to triage THA patients to hospital or video consultation 6 weeks postoperatively. As the main finding, a triage tool based on PROM questions measuring moderate or severe pain and whether the patient used 2 crutches was developed. The included questions were NRS pain during activity; EQ-5D-3L questions 1 and 4; and OHS questions 6, 8, and 12. Applying the final triage tool in both the training and test cohorts resulted in the same outcome: Around 70% of the patients could safely have a video consultation, and 30% needed to have a hospital consultation 6 weeks postoperatively. Therefore, this PRO triage tool is a promising instrument to select patients for video consultation while using an existing PROM infrastructure. The next step is to further investigate this triage tool in daily practice.

This study showed that 70% of the hospital consultations for THA patients 6 weeks postoperatively could safely be done by video. It was hypothesized that 10% of the THA patients would need a hospital consultation. First, the result that 30% of patients needed a hospital consultation could be explained by the focus of the clinical expert panel. As the experts’ beginning point was seeing all patients during a hospital consultation (100%), by developing the triage tool, they desired to see all patients who potentially needed a physical examination or other examination, such as an X-ray, during a hospital consultation. Furthermore, they desired to prevent obtaining more consultations by needing to schedule a hospital consultation after a video consultation. Both implicitly resulted in more liberal criteria for a hospital consultation leading to more patients triaged to a hospital consultation. Second, it could be that specific questions are missing from the triage tool. It was hypothesized that, after an investigation of the triage tool in daily practice, the criteria for the triage tool could be improved, achieving the right health care for each patient and a further reduction in hospital consultations. It would be interesting to investigate how many additional hospital consultations would be needed if the tool triages to video consultation and which PROs are different for patients with an additional hospital consultation.

It is essential to understand that the PRO triage tool is not a tool on its own, but it is the first step in the selection of patients who need a hospital consultation and those who can have a video consultation instead. PROs and clinical judgment produce complementary data and when combined, provide a more accurate description of the patients’ symptoms [24]. Therefore, using the current PRO triage tool, clinicians should have the ability to change the outcome of the tool. To further develop the triage tool, it would be interesting to investigate how many times clinicians decide to change the outcome and which PROs are different for the patients whose clinicians decide to change the outcome. Furthermore, it would be interesting to take the patient’s preference into account.

Previous studies reported that patients rate their video consultations as excellent or very good (92%-95%) [25,26]. The patient no-show rate has been reported at 2.8%, and their mean estimated saved travel time is 30 minutes [25]. Furthermore, 82% would recommend video consultation to family and friends [26]. Almost all clinicians rate their video consultation experience as very good or excellent (92%). They are comfortable with executing this type of consultation after 1 to 4 sessions (69%) [25]. Therefore, video consultation is a serious alternative for hospital consultation. Numbers could be improved when appropriate patients for video consultation are selected, which makes the developed PRO triage tool a promising instrument.

As a first strength of this study, to the authors’ knowledge, this is the first study in which a tool to triage patients to hospital or video consultation was developed. Second, high response rates preoperatively and even postoperatively (above 90%) were achieved, resulting in a representative cohort to execute this study and to generalize the results to the total THA population. A third strength is the application of the training and test cohorts to provide an unbiased evaluation of the final tool.

As a limitation of this study, the triage tool was not investigated in a prospective cohort, and aspects of reliability, validity, sensitivity, and specificity of the triage tool were not investigated yet. Furthermore, 3-month PROs were used instead of 6-week PROs, as, although based on previous studies, the assumption was made that there is limited clinically relevant difference between PROs at 6 weeks and at 3 months postoperatively [11,12]. Future research should be executed in a prospective cohort, and aspects of reliability, validity, sensitivity, and specificity need to be investigated to further develop the THA PRO triage tool. The triage tool could be improved by investigating which PROs are different for patients with additional hospital consultations after being triaged to video consultation or for patients whose clinician decided to change the outcome of the triage tool. After improving the triage tool, it would be interesting to investigate if and which of the patients triaged to video consultation may not require a consultation at all.

In conclusion, a THA PRO triage tool based on moderate or severe pain and using 2 crutches was developed. Around 70% of THA patients could safely have a video consultation, and 30% of patients needed a hospital consultation 6 weeks postoperatively. This tool is promising for selecting patients for video consultation while using an existing PROM infrastructure.

## Abbreviations

ASA: American Society of Anesthesiologists
EQ VAS: EuroQol visual analogue scale
EQ-5D descriptive system: EuroQol 5 dimensions descriptive system
EQ-5D-3L: 3-level version of the EuroQol 5 dimensions
HOOS-PS: Hip disability and Osteoarthritis Outcome Score – Physical Function Shortform
NRS: numeric rating scale
OHS: Oxford Hip Score
PROM(s): patient-reported outcome measure(s)
PRO(s): patient-reported outcome(s)
THA: total hip arthroplasty

## References

[1] Merkes K, Hagenaars N (2020). Corona: katalysator of struikelblok voor groenere ziekenhuiszorg?. Gupta Strategists.

[2] Hollander Judd E, Carr Brendan G (2020). Virtually Perfect? Telemedicine for Covid-19. N Engl J Med.

[3] O'Connor CM, Anoushiravani AA, DiCaprio MR, Healy WL, Iorio R (2020). Economic recovery after the COVID-19 pandemic: resuming elective orthopedic surgery and total joint arthroplasty. J Arthroplasty.

[4] Kane LT, Thakar O, Jamgochian G, Lazarus MD, Abboud JA, Namdari S, Horneff JG (2020). The role of telehealth as a platform for postoperative visits following rotator cuff repair: a prospective, randomized controlled trial. J Shoulder Elbow Surg.

[5] Rolfson O, Bohm E, Franklin P, Lyman S, Denissen G, Dawson J, Dunn J, Eresian Chenok K, Dunbar M, Overgaard S, Garellick G, Lübbeke A, Patient-Reported Outcome Measures Working Group of the International Society of Arthroplasty Registries (2016). Report of the Patient-Reported Outcome Measures Working Group of the International Society of Arthroplasty Registries Part II. Recommendations for selection, administration, and analysis. Acta Orthop.

[6] PROMs. Nederlandse Orthopaedische Vereniging.

[7] Palazzo C, Jourdan C, Descamps S, Nizard R, Hamadouche M, Anract P, Boisgard S, Galvin M, Ravaud P, Poiraudeau S (2014). Determinants of satisfaction 1 year after total hip arthroplasty: the role of expectations fulfilment. BMC Musculoskelet Disord.

[8] Anakwe RE, Jenkins PJ, Moran M (2011). Predicting dissatisfaction after total hip arthroplasty: a study of 850 patients. J Arthroplasty.

[9] Hesseling B, Mathijssen NMC, van Steenbergen LN, Melles M, Vehmeijer SBW, Porsius JT (2019). Fast starters, slow starters, and late dippers: trajectories of patient-reported outcomes after total hip arthroplasty: results from a Dutch nationwide database. J Bone Joint Surg Am.

[10] (2019). Routinematige follow-up bij THP - Richtlijnendatabase. Federatie Medisch Specialisten.

[11] Klapwijk LCM, Mathijssen NMC, Van Egmond JC, Verbeek BM, Vehmeijer SBW (2017). The first 6 weeks of recovery after primary total hip arthroplasty with fast track. Acta Orthop.

[12] Hartog YMD, Mathijssen NMC, Vehmeijer SBW (2015). Total hip arthroplasty in an outpatient setting in 27 selected patients. Acta Orthop.

[13] Pronk Y, Pilot P, Brinkman JM, van Heerwaarden RJ, van der Weegen W (2019). Response rate and costs for automated patient-reported outcomes collection alone compared to combined automated and manual collection. J Patient Rep Outcomes.

[14] EuroQol Group (1990). EuroQol–a new facility for the measurement of health-related quality of life. Health Policy.

[15] Davis AM, Perruccio AV, Canizares M, Hawker GA, Roos EM, Maillefert J, Lohmander LS (2009). Comparative, validity and responsiveness of the HOOS-PS and KOOS-PS to the WOMAC physical function subscale in total joint replacement for osteoarthritis. Osteoarthritis Cartilage.

[16] Davis AM, Perruccio AV, Canizares M, Tennant A, Hawker GA, Conaghan PG, Roos EM, Jordan JM, Maillefert J, Dougados M, Lohmander LS (2008). The development of a short measure of physical function for hip OA HOOS-Physical Function Shortform (HOOS-PS): an OARSI/OMERACT initiative. Osteoarthritis Cartilage.

[17] Gosens T, Hoefnagels NHM, de Vet RCW, Dhert WJA, van Langelaan EJ, Bulstra SK, Geesink RGT (2005). The "Oxford Heup Score": the translation and validation of a questionnaire into Dutch to evaluate the results of total hip arthroplasty. Acta Orthop.

[18] Terwee CB, Bot SDM, de Boer MR, van der Windt DAWM, Knol DL, Dekker J, Bouter LM, de Vet HCW (2007). Quality criteria were proposed for measurement properties of health status questionnaires. J Clin Epidemiol.

[19] Streiner DL, Norman GR, Cairney J, Streiner DL, Norman GR, Cairney J (2019). Reliability. Health Measurement Scales: A practical guide to their development and use.

[20] Terwee CB, Dekker FW, Wiersinga WM, Prummel MF, Bossuyt PMM (2003). On assessing responsiveness of health-related quality of life instruments: guidelines for instrument evaluation. Qual Life Res.

[21] Moayyedi P, Duffett S, Braunholtz D, Mason S, Richards ID, Dowell AC, Axon AT (1998). The Leeds Dyspepsia Questionnaire: a valid tool for measuring the presence and severity of dyspepsia. Aliment Pharmacol Ther.

[22] Pinto PR, McIntyre T, Ferrero R, Almeida A, Araújo-Soares V (2013). Risk factors for moderate and severe persistent pain in patients undergoing total knee and hip arthroplasty: a prospective predictive study. PLoS One.

[23] Breivik H, Borchgrevink PC, Allen SM, Rosseland LA, Romundstad L, Hals EKB, Kvarstein G, Stubhaug A (2008). Assessment of pain. Br J Anaesth.

[24] Basch E, Bennett A, Pietanza MC (2011). Use of patient-reported outcomes to improve the predictive accuracy of clinician-reported adverse events. J Natl Cancer Inst.

[25] Tenforde AS, Iaccarino MA, Borgstrom H, Hefner JE, Silver J, Ahmed M, Babu AN, Blauwet CA, Elson L, Eng C, Kotler D, Homer S, Makovitch S, McInnis KC, Vora A, Borg-Stein J (2020). Telemedicine during COVID-19 for outpatient sports and musculoskeletal medicine physicians. PM R.

[26] Donelan K, Barreto EA, Sossong S, Michael C, Estrada JJ, Cohen AB, Wozniak J, Schwamm LH (2019). Patient and clinician experiences with telehealth for patient follow-up care. Am J Manag Care.

